# Altered somatosensory processing in adult attention deficit hyperactivity disorder

**DOI:** 10.1186/s12888-024-06002-9

**Published:** 2024-08-13

**Authors:** Morgan Frost-Karlsson, Andrea Johansson Capusan, Håkan Olausson, Rebecca Boehme

**Affiliations:** 1 Center for Social and Affective Neuroscience, Department of Biomedical and Clinical Sciences, Linköping University, Linköping, Sweden; 2 Department of Psychiatry in Linköping, Department of Biomedical and Clinical Sciences, Linköping University, Linköping, Sweden; 3 Department of Clinical Neurophysiology, Linköping University Hospital, Linköping, Sweden; 4 Department of Medical Imaging Visualization (CMIV), Linköping University, Linköping, Sweden; 5 Department of Clinical and Experimental Medicine, Center for Social and Affective Neuroscience (CSAN), Linköping University Hospital, Tinnerbäckshuset Floor 14, Linköping, S-581 83 Sweden

**Keywords:** ADHD, Tactile sensitivity, Sensory gating, Somatosensory processing

## Abstract

**Background:**

Tactile sensitivity and sensory overload in ADHD are well-documented in clinical-, self-, and parent- reports, but empirical evidence is scarce and ambiguous and focuses primarily on children. Here, we compare both empirical and self-report tactile sensitivity and ADHD symptomatology in adults with ADHD and neurotypical controls. We evaluate whether tactile sensitivity and integration is more prevalent in ADHD and whether it is related to ADHD symptom severity.

**Methods:**

Somatosensory evoked potential (SEP) amplitudes were measured in 27 adults with ADHD and 24 controls during four conditions (rest, stroking of the own arm, stroking of the arm by a researcher, and stroking of an object). Participants also filled out questionnaires on tactile sensitivity and ADHD symptoms and performed a Qb-test as an objective measure of ADHD symptom severity.

**Results:**

Participants with ADHD self-reported greater tactile sensitivity and ADHD symptom severity than controls and received higher scores on the Qb-test. These values correlated with one another. ADHD participants showed lower tolerable threshold for electrical radial nerve stimulus, and greater reduction in cortical SEP amplitudes during additional tactile stimuli which was correlated with ADHD symptoms.

**Conclusions:**

We find that ADHD symptomatology and touch sensitivity are directly linked, using both self-reports and experimental measures. We also find evidence of tactile sensory overload in ADHD, and an indication that this is linked to inattention specifically. Tactile sensitivity and sensory overload impact the functioning and life quality of many people with ADHD, and clinicians should consider this when treating their patients.

**Supplementary Information:**

The online version contains supplementary material available at 10.1186/s12888-024-06002-9.

## Introduction

Attention Deficit Hyperactivity Disorder (ADHD) is characterized by inattention, hyperactivity and/or impulsivity [1]. Classified as a neurodevelopmental disorder, it is pervasive in children (5–15% globally) and about half of these diagnoses persist into adulthood [2]. Despite the prevalence of ADHD in the adult population and the significant impact it can have on an individual’s functioning, clinical and empirical research has focused mostly on children and adolescents.

On ADHD popular science media and self-help sites, sensory sensitivity in ADHD is a widely discussed topic. However, scientific evidence on altered sensory processing is scarce, focused mainly on children, and mostly relies on clinical-, self-, or parent- reports Touch-sensitivity affects around one in six children with ADHD [3] and parent- and clinical reports show more sensory difficulties in ADHD than in typically developing children [4, 5]. Some studies on adults with ADHD report greater sensory sensitivity compared to neurotypical controls across all sensory modalities including the tactile sense [6, 7]. Also in the general adult population, self-reports show that more ADHD traits are associated with more sensory difficulties [8].

Among the senses, the tactile sense is the earliest to develop and plays a crucial role for learning about both the world around us and our own body. Through early tactile sensations, even prenatally, a baby can begin to differentiate between the self and the outside world, including other human beings and future interaction partners [9]. Since the sense of touch is so important for social development and learning about the physical world [10–13], alterations in tactile processing might affect have far-reaching and long-lasting effects: they might even causally relate to social interaction difficulties seen in ADHD as well as to typical ADHD symptoms like inattention and hyperactivity [14].

Social difficulties, including understanding social information, attuning behavior to different social contexts, and reduced social contact and/or interest, are often apparent in both children and adults with ADHD. Some of these (e.g. “interrupting or intruding on others”) are included in DSM-5 diagnostic criteria [1], while others, though not diagnostic criteria, clearly emerge from observational studies (e.g., more noncompliance and/or aggression with peers in children [15, 16]). Rather than due to social disinterest, Nijmeijer et al. [17] postulate social dysfunction in children with ADHD to be a consequence of the underlying core ADHD symptoms of hyperactivity, impulsivity, and inattention, as well as of the co-occurring psychiatric and/ or neurodevelopmental conditions common in children with ADHD such as Autism Spectrum Condition, Oppositional Defiant Disorder, and Conduct Disorder, which are more defined by pervasive social impairments.

Only a few empirical studies evaluated tactile processing in ADHD experimentally and the results are ambiguous. In children, Mangeot et al. [5] show physiological abnormalities when collapsing all sensory modalities. Another study in children finds slower reaction times and higher thresholds for tactile perception in ADHD than typically developing children – though the researchers attribute this to inattention and lower cognitive functioning [18]. One study finds that somatosensory evoked potential (SEP) amplitudes are higher in boys with ADHD than typically developing boys at the cortical but not the cervical level [19], indicating an altered higher order processing not present at the spinal cord level. Their follow-up study finds that children with co-occurrence of ADHD and Tactile Defensiveness (a pattern of hyper-sensitive responses to ordinary tactile stimulation) have highest cortical SEP amplitudes, while those with ADHD without Tactile Defensiveness have medium cortical amplitudes (though still higher amplitudes than typically developing children) [20]. In adults, differences in tactile processing have only been investigated in very few studies. Our previous study finds that adults with ADHD do not differ significantly from control participants with regard to thresholds for touch perception [21]. Using functional magnetic resonance imaging however, we find increased activation in somatosensory cortex to social touch, in addition to self-reported social touch aversion. In line with this, brain areas associated with sensory processing are suggested to be functionally altered in ADHD [22].

Our previous magnetic resonance imaging study [21] finds, in adults with ADHD compared to control subjects, a greater activation in somatosensory cortex during skin-to-skin touch by another person and larger deactivation in insula during self-touch. Tactile sensations produced by others are considered socially relevant and are salient, whereas self-generated tactile sensations are attenuated through an efference copy [23]. The efference copy is thought to be implicated in the detection of unpredictable sensations which is important for sensing both danger and reward. Accurate and implicit distinction between self-touch and touch by others is foundational to social interactions and the development of a bodily self [9]. Furthermore, we previously found no difference in tactile detection thresholds between adults with ADHD and neurotypical adults, suggesting intact peripheral tactile processing. It remains unclear at which processing stage the signaling of tactile sensations is altered in ADHD. It further has not been investigated whether altered tactile processing is linked to symptom severity regarding hyperactivity and inattention.

Therefore, our current study used somatosensory evoked potentials (SEP) in a population of adults with ADHD and age- and gender-matched neurotypical adults to expand on our previous findings with regard to three aspects: (1) on differential self- and other-touch processing, (2) on the relationship of these measures to symptomatology, and (3) by adding an exploration of whether such differences occur already at the spinal cord level, or whether the alterations occur more centrally.

Participants performed self-touch and received touch by the experimenter on the arm during SEP recording, performed a Qb-test, an experimental, validated measure of inattention and hyperactivity, and filled in questionnaires regarding ADHD symptom severity and touch sensitivity. Given our and others’ earlier results, we hypothesized (1) greater differences between SEP amplitudes measured during self- and other-touch in ADHD compared to neurotypical controls. We further hypothesized (2) that increased SEP amplitudes should relate to symptom severity obtained via self-reports and via Qb-test. Finally, (3) we hypothesized greater touch sensitivity in the ADHD group as indicated by larger baseline SEP amplitudes and higher scores on the self-report tactile questionnaire. Following up on our previous findings of intact thresholds but altered cortical processing, we also wanted to explore whether we already see altered SEP at spinal cord level (3).

## Methods

### Participants

The study was approved by the ethics committee (2016-360-31, 2017-320-32, 2020–03015). ADHD participants (*n* = 27; mean age 25.2; SD 4.95; range 18–35; 16 female; ) were recruited from the adult psychiatric clinic at the local hospitals and through online advertisements. All participants had been diagnosed with ADHD by their treating psychiatrist according to standard clinical procedure. Exclusion criteria included: any co-occurring current psychiatric disorder (such as, but not exclusively, psychosis, bipolar disorder, OCD), autism spectrum disorder, alcohol or substance use disorder within the past year, chronic pain, or any other major health concern. Participants refrained from taking stimulant medication for 24 h prior to participation (*n* = 10 participants regularly took stimulant medication, which included such as lisdexamphetamin or methylphenidate, for a full medication overview see Supplementary Table S1). Matched neurotypical participants (NT) (*n* = 24; mean age 25.3¸SD 4.83; range 19–35; 14 female; ) were recruited from online advertisements. Exclusion criteria were assessed by a nurse during a telephone interview and included: any psychiatric disorder, alcohol or substance use disorder, chronic pain, or any other major health concern. During the visit, all participants filled in questionnaires pertaining to ADHD symptoms (ASRS, [24]), and tactile sensitivity (based on the tactile questions from the sensory perception quotient [25] and from sensory profile [26], see supplement). Scores were compared using two-sample t-tests. Qb test scores are missing from two ADHD participants who did not complete the test and one neurotypical participant where data was lost due to technical error. Questionnaire data is missing from four ADHD participants and four neurotypical participants due to incomplete questionnaires (some questions were left unanswered and/or the questionnaire was left incomplete by the participant).

#### Somatosensory evoked potentials

After two baseline recordings, SEPs were recorded simultaneously with three touch conditions consisting of slow, light stroking as reported previously [27]: (1) self-touch, during which participants stroked their own left forearm with the right hand, (2) object-touch, where participants stroked a pillow with the right hand (control condition), and (3) other-touch, where participants were stroked on the left forearm by the experimenter. The order of conditions was randomized, and each condition was repeated twice. The area stroked on the left forearm corresponded to innervation by the radial nerve. The stroking occurred simultaneously with the electrical pulse for the whole duration of each run (300 pulses). Participants sat in a reclining seat and were asked to close their eyes, recline, and relax as much as possible to avoid muscle artifacts. The experimenter sat on a chair, next to the participant for the duration of the experiment. A stimulating electrode was placed at the base of the left thumb to stimulate the radial nerve. Stimulus intensity was individually adjusted according to the highest level tolerable for the participant. According to a standard clinical neurophysiology protocol, 300 non-painful pulses at a maximum of 100 mA (range for this sample 7.25–15.1 mA) at 1 Hz were administered, resulting in a length of approximately 3 min per condition. Recording electrodes were placed on the C6 cervical level, and on C4, Cz, and Fz scalp positions. Electrode skin impedance was always less than 8 kΩ. Data acquisition, filtering, and initial analysis was performed on a clinical machine in a hospital clinic, using processes that are automated for clinical use. Data were acquired for 100 ms after each pulse using a Nicolet EDX system with an AT2 + 6 amplifier (Carefusion, Middelton, WI53562, USA), and recorded and analyzed in a standard automated process using Synergy 20.0 (Carefusion, Middelton, WI53562, USA). Recordings were referenced to Fz and bandpass filtered (2 Hz – 2 kHz), amplifier range was 5mV and display sensitivity was 20µV per division. Waveforms were averaged over 300 pulses for each recording electrode and over the two runs per condition, and analyzed regarding amplitude (N15 cervically, N20 cortically). Examples can be found in the supplement (Fig S1). Baseline to peak amplitude was calculated automatically with the baseline defined as the value right before the averaged waveform and with automatically selected peaks, which were inspected individually and manually adjusted if detected incorrectly by the algorithm. Values were compared using repeated measures ANOVA.

### Qb test

The QbTest (QbTech Ltd, www.qbtech.com) uses an infrared camera and reflective headband to monitor a participant’s head movements while they perform a 20-minute button-press task, based on the continuous performance test on a computer. Participants are presented with two different shapes (circle, square), one at a time in either of two different colors (red, blue). The participant is instructed to press a button when the same shape and color appear on the screen twice in a row. The test records parameters such as commission errors, anticipatory responses, and reaction time, while the camera monitors movement time, area, and distance. The scores are compared to a standardized, sex and age matched group from the general population, then transformed and presented in the test reports as a “Q-score” with percentiles. Q-scores consist of three main parameters (QbInattention, QbActivity, QbImpulsivity) corresponding to the three main symptom domains of ADHD: inattention (measured through reaction time and omission errors), hyperactivity (measured through movements), and impulsivity (measured through reaction time and commission errors). Higher Q-scores (especially above 1.5) generally indicate more severe ADHD symptoms. In this study, we calculated a sum of Qb scores as an indicator of symptom severity.

## Results

### Demographics

**Fig. 1:**
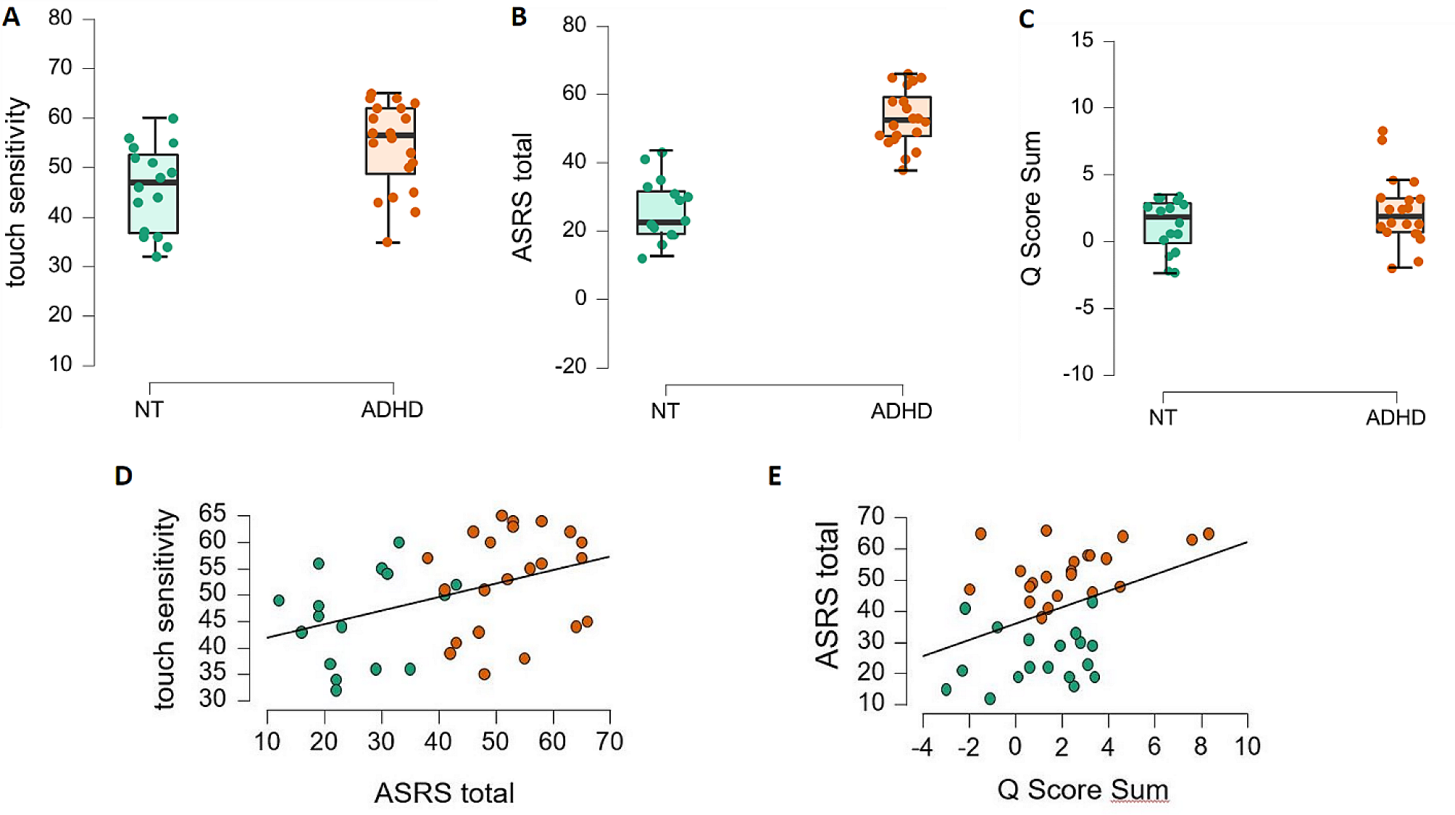
Differences between NT control (green) and ADHD (orange) groups for (**A**) touch sensitivity, (**B**) Adult ADHD Self-Report Scale (ASRS), and (**C**) Q score sums; correlations across groups between ASRS and (**D**) touch sensitivity and (**E**) Q score sums

Participant characteristics are presented in Table 1. The ADHD group showed higher self-reported touch sensitivity than the NT control group (t = -2.59 *p* = 0.013, Fig. 1A), as well as higher self-report ADHD symptom severity, measured through ASRS as a total score (t = -10.29, *p* < 0.001, Fig. 1B), with subscales for inattention (t = -10.05, *p* < 0.001) and hyperactivity (t = -7.98, *p* < 0.001). ADHD also had higher Q-score sums (t = -2.18, *p* = 0.034, Fig. 1C). Self-report touch sensitivity correlated positively with ASRS across groups (*r* = 0.419, *p* = 0.009, Fig. 1D). Q-score sums also correlated with ASRS scores (*r* = 0.388, *p* = 0.013, Fig. 1E). These correlations were not significant within group (Supplementary Table S3).

**Table 1:** Characteristics of adults with ADHD and neurotypical control (NT) participants

	NT	ADHD	*p*-value
age (m, *SD*)	25.32 *(4.83)*	25.19 *(4.95)*	0.921
sex (N, %)	f:14 (58%) m: 10 (42%)	f: 16 (59%) m: 11 (41%)	0.958
Qb sum (m, *SD*)	0.88 *(2.12)*	2.41 *(2.7)*	0.034
ASRS total (m, *SD*)	26.11 *(8.99)*	52.64 *(8.07)*	0.009
ASRS inattention (m, *SD*)	15.47 *(5.33)*	29.96 *(4.24)*	< 0.001
ASRS hyperactivity (m, *SD*)	10.79 *(5.05)*	22.12 *(4.35)*	< 0.001
Tactile sensitivity (m, *SD*)	45.17 *(8.49)*	52.42 *(9.33)*	0.013

m: mean; SD: standard deviation; NT: neurotypical controls; ASRS: Adult ADHD Self-Report Scale; Qb: sum of the scores from the Qb-test; p-values obtained from two sample t-tests

### SEP amplitudes

When comparing baseline C4 amplitudes, though there was a trend there was no significant difference between groups (mean ADHD ± SD = 3.36 ± 1.12, mean NT ± SD = 2.81 ± 0.8, t = -1.97, *p* = 0.055, Fig S2). Stimulus intensity (individually determined based on highest tolerable level), however, was significantly higher in NT (mean ADHD ± SD = 9.74 ± 1.46, range 7.25–12.5; mean NT ± SD = 10.87 ± 1.85, range 7.4–15.1, t = 2.442, *p* = 0.018, Fig S2). When adjusting for stimulus intensity, baseline C4 amplitudes differed between groups (mean ADHD ± SD = 0.351 ± 0.127, mean NT ± SD = 0.262 ± 0.076, t=-3.043, *p* = 0.004). There was no relationship between individually adjusted intensity and C4 amplitude (*r* = 0.07, *p* = 0.621, Fig S3). We therefore corrected all following analyses for individual baseline amplitudes.

**Fig. 2:**
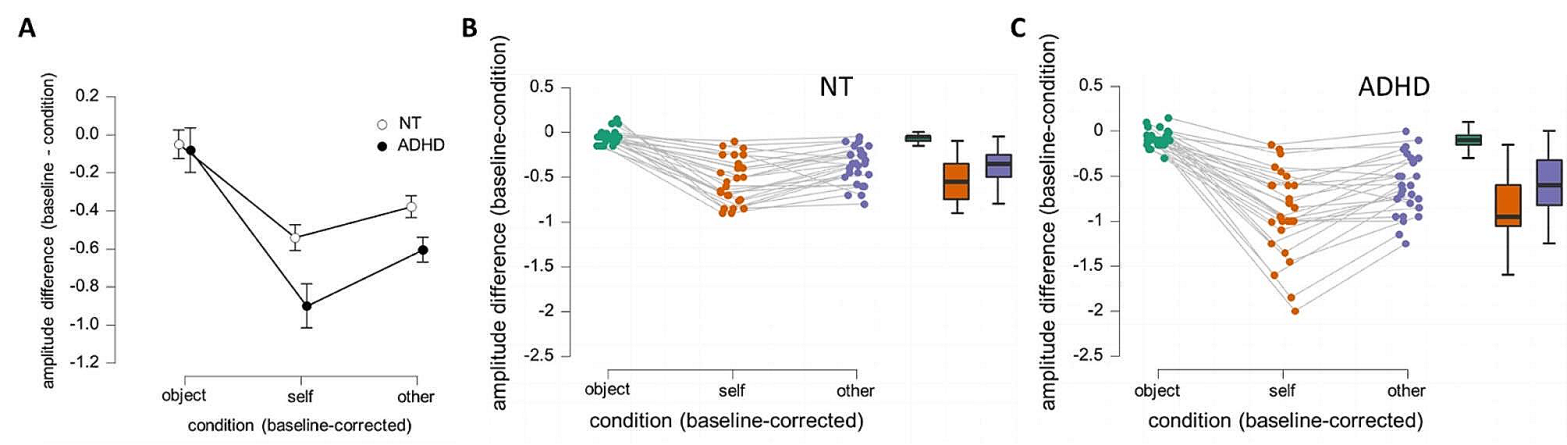
ADHD participants show larger amplitude decreases at the cortical level. (**A**) Overall amplitude differences between groups and conditions. **B**, **C**) Differences between conditions in the (**B**) NT control group and (**C**) ADHD group

Repeated measures ANOVA showed a main effect of condition (F = 123.872, *p* < 0.001) as well as a group*condition interaction (F = 7.499, *p* < 001, Fig. 2). Post-hoc comparisons revealed group differences for the self-touch condition (mean ADHD ± SD = -0.9 ± 0.473, mean NT ± SD = -0.541 ± 0.254, t= -3.379, *p* = 0.001) and the other-touch condition (mean ADHD ± SD = -0.604 ± 0.327, mean NT ± SD = -0.379 ± 0.205, t = -3.076, *p* = 0.003), but not for the object-touch condition (mean ADHD ± SD = -0.082 ± 0.097, mean NT ± SD = -0.05 ± 0.077, t = -1.286, *p* = 0.204). A Levene’s Test of Equality of Variance showed larger variation in amplitude differences in the ADHD group for both self (F = 5.770, *p* = 0.02) and other (F = 5.438, *p* = 0.024) conditions.

There was an overall difference across groups between self- and other-conditions (t = 6.31, *p* < 0.001). There were no differences between groups for the other-vs- self-conditions (t=-1.650, *p* = 0.105). No differences were found between conditions (F = 0.886, *p* = 0.416) or groups (F = 0.904, *p* = 0.408) at the cervical level (Figure S4).

### Correlations with symptom severity

Individually adjusted stimulus intensity and self-reported touch sensitivity showed a negative correlation across groups (*r* = − 0.425, *p* = 0.004). Touch sensitivity also correlated negatively with baseline corrected C4 amplitudes for the self- (*r* = -0.384, *p* = 0.012) and other- (*r* = − 0.322, *p* = 0.038) conditions across groups, but not within groups (Supplementary Table S3). And likewise, ASRS total scores correlated negatively with baseline corrected C4 amplitudes for the self- (*r* = -0.431, *p* = 0.003) and other- (*r* = -0.342, *p* = 0.023) conditions.

Considering our specific hypotheses that inattention and hyperactivity might be related to sensory processing alterations, we also explored correlations with subscores across groups: ASRS inattention subscores correlated negatively with baseline corrected C4 amplitudes for the self- (*r* = -0.405, *p* = 0.006) and other- (*r* = 0.333, *p* = 0.026) conditions. Hyperactivity subscores correlated weakly with the amplitude during the self- (*r*= -0.3, *p* = 0.048) but not with the amplitude during the other- (*r*=-0.282, *p* = 0.064) condition.

No correlations were found between Q score sums and C4 amplitude differences for either the self- (*r*=-0.237, *p* = 0.104) or other- (*r*=-0.24, *p* = 0.101) conditions. No correlations were found for Qb-subscores (Supplementary table S2).

## Discussion

Our study evaluated somatosensory evoked potentials in adults with ADHD and found evidence for altered integration of tactile stimuli, which was associated with self-reported symptom severity.

We found differences between adults with an ADHD diagnosis and neurotypical matched control participants regarding self-reported ADHD symptom severity, touch sensitivity, and experimental Q-score. Self-reported touch sensitivity was associated with self-reported symptom severity across groups, but not within groups. Differences in symptom severity and Q-scores were unsurprising given that the participants refrained from taking stimulant medication before the study. Our findings on touch sensitivity replicated previous findings of self-, clinician-, and parent-reported tactile sensitivity in ADHD (see, e.g., [4, 7, 14]). Touch sensitivity was correlated with ADHD symptom severity across groups, indicating that the two are specifically linked, which is in line with a self-report study of 274 non-clinical adults indicating a correlation between ASRS score and sensory sensitivity (determined via the Highly Sensitive Person Scale) in the general population [8]. It is important to point out that we found these relationships only in correlations across groups, where they might be driven by between-group differences. However, there was a clear overlap between groups for the Qb-scores (the objective measure), while the self-reported scores differed more distinctly between groups. ADHD traits are found in the general population and it is suggested that ADHD in its clinical presentation represents individuals who are found on the far end of the distribution [28, 29]. We did not find significant within-group correlations. This might be due to the relatively smaller group size and smaller variance within the two groups and needs to be explored in a larger sample size.

We found differences for both group and condition in somatosensory cortical amplitudes, with highest amplitudes overall during rest, reduced amplitudes for the social touch condition, and lowest amplitudes during self-touch (and no difference during the object-touch control condition). This replicates previous SEP results in a healthy population [27] and shows a clear distinction at the cortical level between self-produced and other-produced tactile stimuli. Contrary to our hypothesis and previous findings from other evoked potential studies, we did not find a clear difference between groups for baseline cortical amplitude (though there was a trend). However, this might have been due to the between-group difference of the individually adjusted stimulation intensity. Participants with ADHD considered the SEP stimulation more noxious compared to the neurotypical participants. Additionally, the thresholds participants considered “noxious” negatively correlated with self-reported tactile sensitivity, i.e., the more sensitive to touch a participant reported to be in the questionnaire, the lower threshold they considered tolerable. This again was a correlation across groups though and did not survive in the smaller within group samples. Taken together, this provides both self-report and empirical evidence for increased tactile sensitivity in ADHD and validated our touch sensitivity questionnaire.

Due to these differences in thresholds across participants, we corrected amplitudes according to participants’ baseline amplitudes. We found differences between groups for SEP amplitudes elicited by electrical pulses simultaneous with self- and other-touch: compared to the neurotypical group, the ADHD group showed a larger amplitude decrease for both touch conditions when the electrical pulse occurred on the same arm. There were no differences between groups for the object-touch condition, indicating that the differences were driven by altered integration of touch stimuli simultaneously occurring on the same arm (same dermatome), and not by either the motor component during self-touch or any additional tactile input on a different body part, such as the tactile input through the hand touching the object in the object-touch condition.

Additionally, we found that self-reported ADHD symptoms correlated with SEP amplitudes during self- and other-touch across groups. More severe self-reported ADHD symptoms corresponded to a greater difference in amplitude between baseline and both self- and other-touch condition. These correlations were driven by the ASRS inattention subscore. A less direct but still significant correlation was observed between self-reported touch sensitivity and amplitude difference for the self-condition, while a trend was observed for the other condition.

Taken together, these findings support a relationship between altered tactile processing and ADHD symptomatology in adults. An explanation could be that the ADHD group experienced sensory overload with both the stimulus and the stroking occurring simultaneously. A combination of attention deficit and somatosensory sensitivity could make it more difficult for people with ADHD to integrate competing tactile sensations, habituating to what is irrelevant and attending to what is relevant; a process often referred to as sensory gating. However, gating, habituation, and attenuation of sensory stimuli are dissociable phenomena describing different underlying mechanisms [30]. We will therefore here refer to the overarching process as ‘sensory focus’. Sensory focus, or the lack thereof, is often reported anecdotally in the clinic, with ADHD patients having trouble ignoring the seams of their socks or the tag of a shirt. Similar sensory focus difficulties in ADHD have been described also in the auditory domain (with, for example, patients showing sensitivity “towards sounds which are unheard by others such as the humming of a refrigerator, a clock ticking, or fans” [4]). Evoked potential studies indicate an effect of auditory sensory focus capacity on attention and executive function in adults with ADHD [31]. Studies on tactile sensory focus in ADHD are inconclusive. While some results indicate enhanced habituation, others show reduced habituation, and given the limited methods employed (mainly skin conductance and heart rate) the results are difficult to interpret [32]. Given that the amplitude differences in our study correlated strongly with the ASRS inattention subscore, and less strongly but still significantly with touch sensitivity scores, sensory focus difficulties seem to be a valid explanation for our results. Inconclusive evidence in the existing ADHD literature could also reflect the heterogeneity of the ADHD population; that individuals with ADHD vary in their experience of sensory stimuli. Indeed, some individuals with ADHD may be over-aroused while others may be under-aroused. This concept is reflected in our finding that variation in amplitude differences was significantly greater in the ADHD group compared to the neurotypical group. A similar result was reported in children with ADHD in a study on sensory modulation [33].

We observed no differences in cervical amplitudes for any condition or between groups, indicating that the altered touch processing we observed is a cortical process. This is in line with a previous study on children with ADHD showing higher cortical amplitudes than typically developing controls but no difference cervically [19]. It also aligns with results from our previous study on adults with ADHD showing intact detection thresholds, as evidence of a more central and higher order alteration of touch perception.

Some limitations need to be considered. The participants with ADHD in our study were young adults with no psychiatric comorbidities, who were able to independently schedule and attend the appointment while remembering to abstain from their medication for 24 h. This arguably excludes a large subset of the ADHD population, given the high comorbidity of ADHD with other neuropsychiatric conditions and the difficulties adults with severe ADHD typically have planning and keeping appointments [34] limiting generalizability of our findings to the more severe end of the ADHD spectrum with multiple comorbidities. Since touch sensitivity correlated with symptoms severity and more specifically inattention measures, it may be speculated that individuals with more severe ADHD could also experience more issues with touch sensitivity and with being distracted or overwhelmed by touch (and other) sensory input. It is also possible that at least some of these issues may be alleviated by adequate medication, however the effects of medication on sensory focus or touch sensitivity have not been studied. Earlier studies on sensory gating mainly in the auditory modalities provided contradictory results [35, 36]. Additionally, we were unable to measure or control for the force of the touch coming from the participants and experimenters. Individual differences in pressure as well as pleasantness and preference could influence how each participant stroked their own arm. This should however not affect the other-touch condition as the trained experimenter applied the same type of touch to all participants. Finally, as discussed above, it is important to point out that the correlations were performed across groups and non-significant within the groups separately. While we believe that these relationships are meaningful considering the presence of ADHD traits within the general population and the model that clinical expressions of ADHD represent an extreme within this distribution [28, 29], they might also be driven by between-group differences. At least one previous study found a relationship between sensory processing difficulties and ADHD traits in the general population [8].

## Conclusions

Our study found a link between ADHD symptomatology and touch sensitivity, using both self-reports and experimental measures. Adults with ADHD had lower tolerance to radial nerve stimulation in addition to greater self-reported tactile sensitivity, and these values were associated with one another. Both self-report and experimentally measured touch sensitivity correlated with self-reported overall ADHD symptom severity. Using SEPs, we found that adults with ADHD showed greater differences in cortical amplitudes relative to baseline compared to a neurotypical control group during both a self-touch condition and touch by another. This could be due to increased sensory load, as indicated by correlations between amplitude differences and both touch sensitivity and self-reported ADHD symptoms. A limitation of our study design is that the association between tactile sensitivity, SEP amplitudes, and overall ADHD symptomatology can only be investigated correlationally. It is therefore not possible to draw conclusions regarding the directionality of this association. Inattention and hyperactivity could contribute to the larger interaction of SEP amplitudes with additional touch stimulation. However, one might also speculate that increased sensitivity to tactile stimuli could lead to inattention and hyperactivity. Future studies should try to understand directionality to develop novel treatment options and better support for people in need. Independent of the mechanistic understanding of directionality, increased touch sensitivity and difficulty integrating tactile sensations may contribute to the day-to-day difficulties that individuals with ADHD experience. Clinicians need to be aware of and help their patients address potential difficulties this may incur.

### Electronic supplementary material

Below is the link to the electronic supplementary material.

Supplementary Material 1

Supplementary Material 2

## Data Availability

The dataset used and analyzed during the current study are available in the supplementary materials.
