# Supplementary material for: Altered somatosensory processing in adult attention deficit hyperactivity disorder
Source: BMC Psychiatry. 2024 Aug 13;24:558. doi: 10.1186/s12888-024-06002-9 (PMC11323665; doi:10.1186/s12888-024-06002-9)
Supplement: Supplementary file 2 — Supplementary Material 2 [file 12888_2024_6002_MOESM2_ESM.docx]

**Supplement**

Altered somatosensory processing in adult attention deficit hyperactivity disorder

Morgan Frost-Karlsson^1^, Andrea Johansson Capusan^1,2^, Håkan Olausson^1,3^, Rebecca Boehme^1, 4^

^1^ Center for Social and Affective Neuroscience, Department of Biomedical and Clinical Sciences, Linköping University, Linköping, Sweden.

^2^ Department of Psychiatry in Linköping and Department of Biomedical and Clinical Sciences, Linköping University, Linköping, Sweden.

^3^ Department of Clinical Neurophysiology, Linköping University Hospital, Linköping, Sweden

^4^ Department of Medical Imaging Visualization (CMIV), Linköping University, Linköping, Sweden

*Methods*:

Tactile Sensitivity Questionnaire based on the tactile questions from the sensory perception quotient and sensory profile, ratings from 1 to 5.

Question from Sensory Perception Quotient, Tavassoli et al.:

1. I wouldn’t be able to feel the label at the back of my shirt even if I thought about it.
2. I would be able to tell the weight difference between two different coin sizes on the palm of my hand, if my eyes were closed.
3. I wouldn’t notice the difference between even and uneven ground when driving over it sitting in the back seat of a car.
4. I wouldn’t be able to feel the vibrations from loud music if I was sitting next to the loud speaker (for example, at a concert).
5. I would be able to feel the elastic holding up my socks if I stop and thought about it.
6. I notice the weight and pressure of a hat on my head.
7. I would feel if a single hair touched the back of my hand.
8. If my mobile phone was vibrating in my pocket I would be quick to sense it.

Questions adjusted from Sensory Profile, Dunn, et al.:

1. Dental work and toothbrushing cause me discomfort.
2. Shoes and Socks irritate me.
3. I do not like standing in line or close to people.
4. People sometimes become irritated with me because I touch them too much.
5. I am a “touchy” person.
6. I am a picky eater - Certain food textures disgust me (e.g. bananas, yoghurt, mushrooms).
7. I avoid certain types of clothing.
8. I avoid certain kinds of materials (rough textures).
9. I cannot wear stiff clothes, clothes with rough textures, turtlenecks, etc.

Table S1. Overview over medications.

| Medication type | Medication | No of participants | Dose [mg/day] |
| --- | --- | --- | --- |
| Stimulants | Oros methylphenidate | 1 | 45 |
|  | Lisdexamphetamine | 5 | 60  60  50  40  40 |
|  | Methylphenidate XR | 2 | 25  120 |
|  | Dexamphetamine | 2 | 15  2-4 |
| Antidepressants | Fluoxetin | 1 | 10 |
|  | Buspiron | 1 | 60 |
|  | Sertalin | 2 | 50  100 |
|  | Bupropion | 1 | 300 |
| Gabapentinoids | Gabapentin | 2 | 1200  1200 |
|  | Pregabaline | 1 | 450 |
| Sleep medication | Melatonin | 2 | 10  8 |
|  | Zopiklon | 1 | 15 |
| Other | Testosterone | 1 | 4 ml every 11^th^ week |
|  | Rivaroxaban | 1 | 20 |
|  | Propranolol | 1 | 40 |
|  | Prometazine | 1 | 25 |

Figure S1: Examples of SEP waveforms. Displayed are the waveform from two runs per conditions. Top: C4; Middle: Cz; Bottom: Cervical. A) Baseline, B) Self, C) Other.


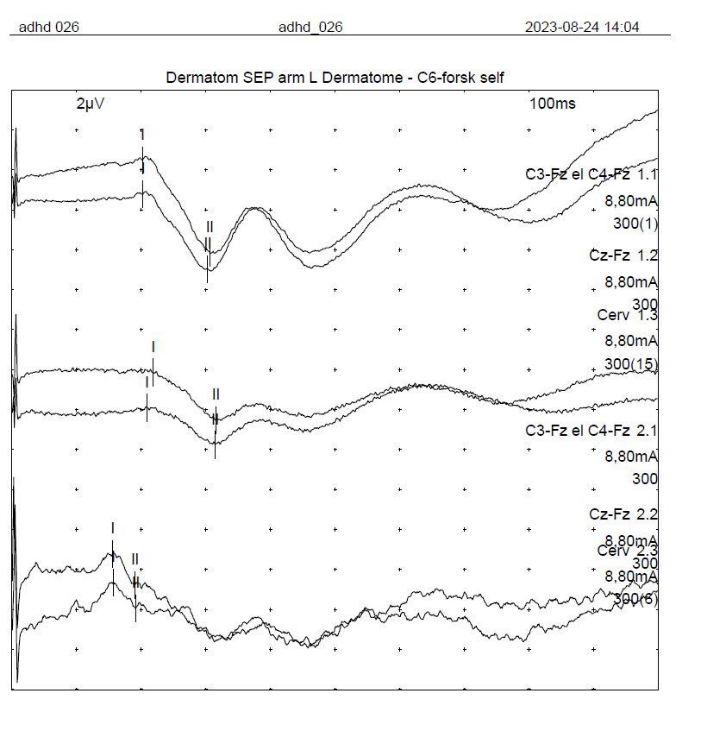

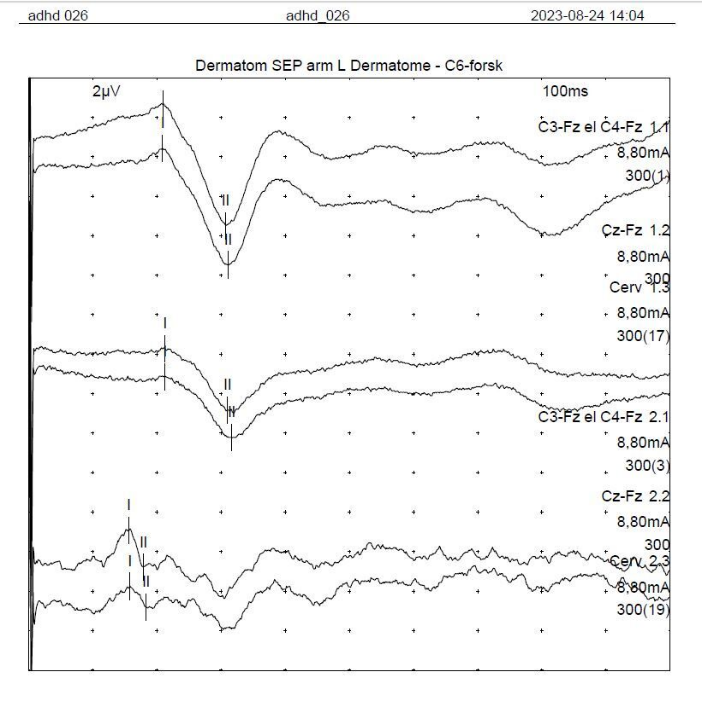
A) B)


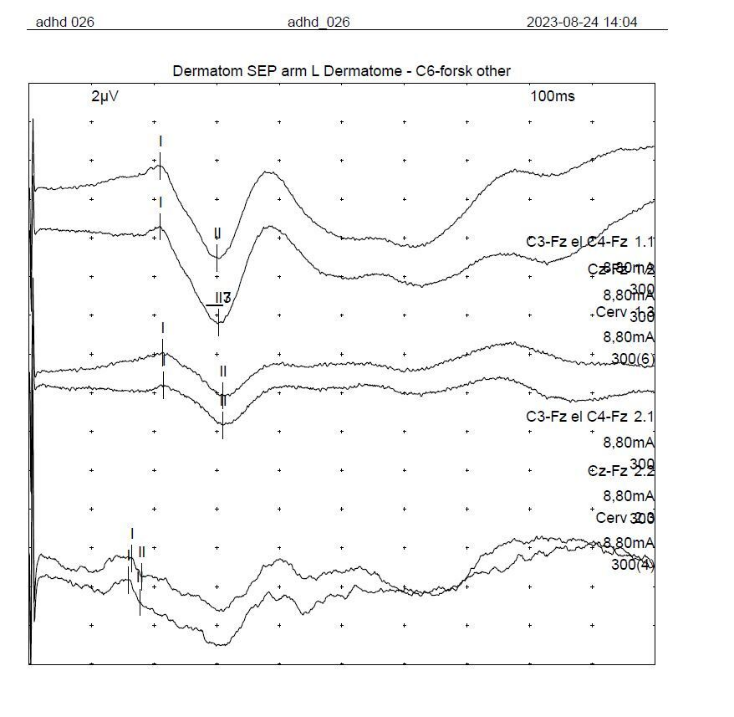
 C)

*Results:*

Figure S2: ADHD and controls did not differ in baseline C4 amplitude (mean ADHD ±SD = 3.36 ± 1.12, mean NT ±SD = 2.81 ± 0.8, t = -1.97, p = 0.055), but differed in the level of radial nerve stimulation that was tolerable (mean ADHD ±SD =9.74 ± 1.46 , mean NT ±SD = 10.87 ± 1.85, t = 2.442, p = .018)


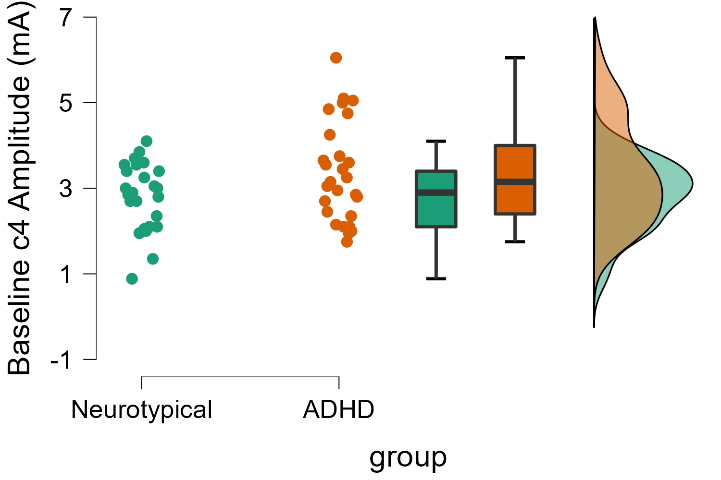

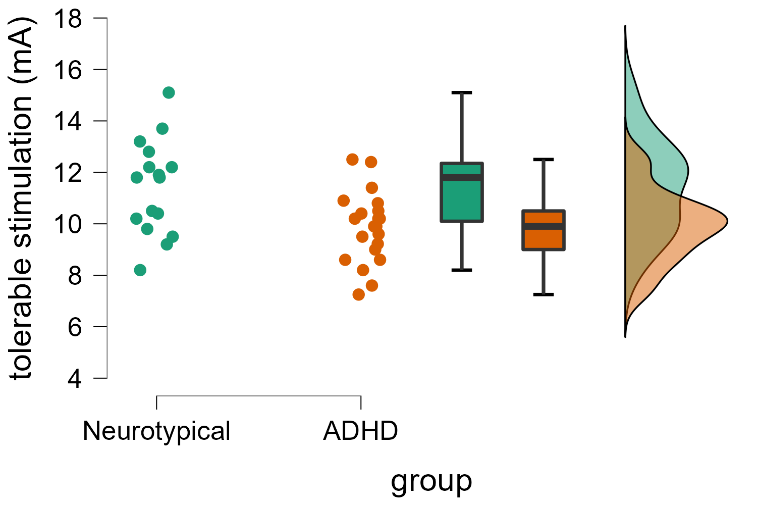


Figure S3: No relationship between individually adjusted stimulus intensity and C4 amplitude.


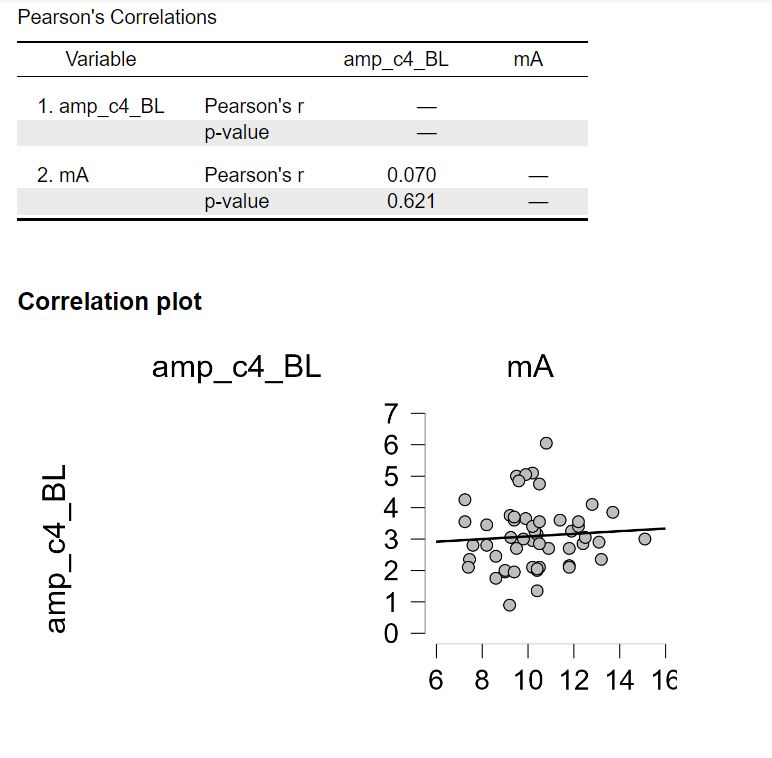


C4 amplitude [mA]

Stimulus intensity [mA]

Figure S4: No differences were found at the cervical level between groups (F = 0.904, p=0.408) or conditions (F = 0.886, p=0.416).


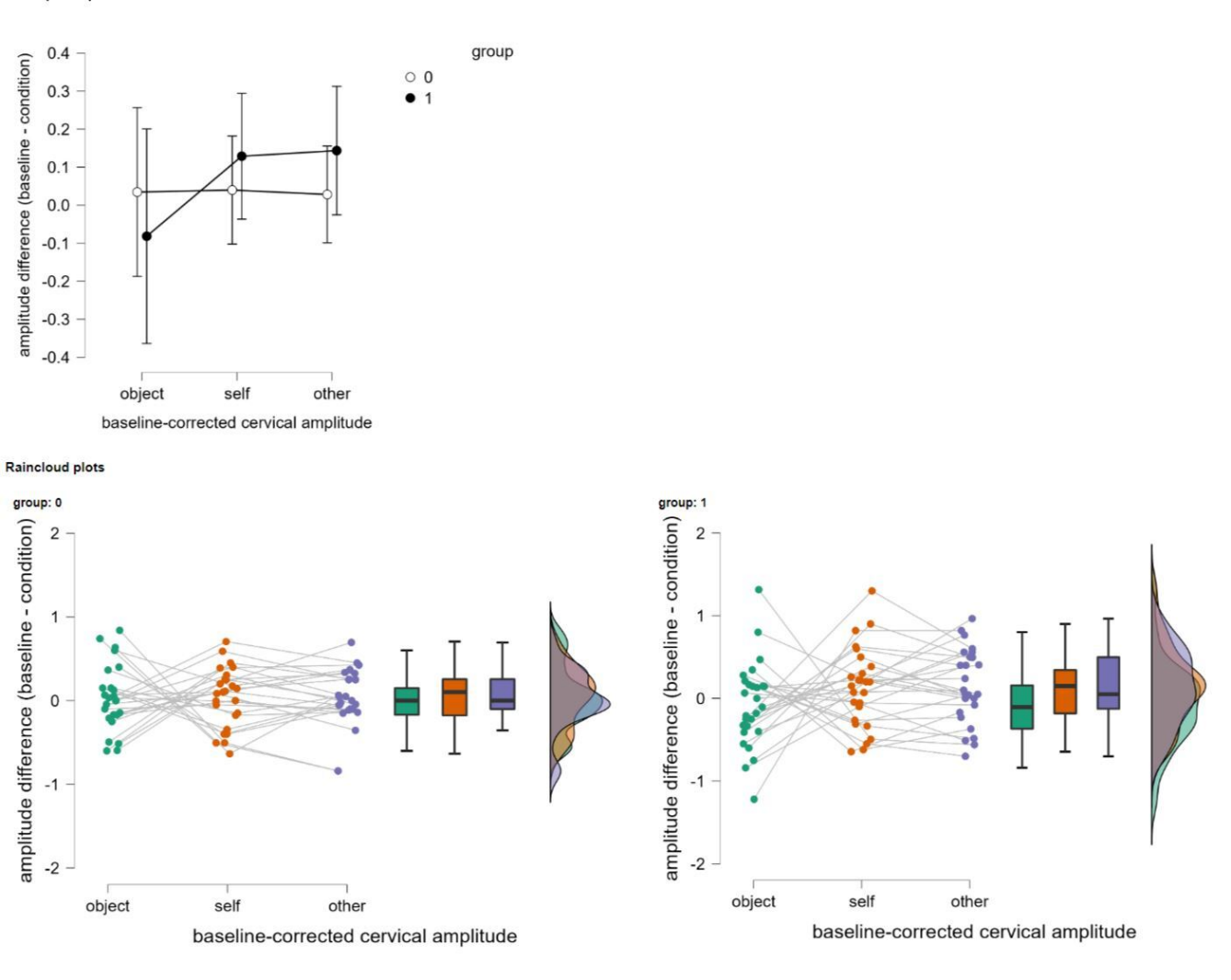


Table S2: Correlations between Qb-subscores and C4 amplitudes during self- and other-conditions.

|  | Self | other |
| --- | --- | --- |
| Q-activity | r=-0.261, p=0.073 | r=-0.247, p=0.09 |
| Q-attention | r=-0.144, p=0.328 | r=-0.247, p=0.09 |
| Q-impulsivity | r=-0.116, p=0.431 | r=-0.013, p=0.928 |

Table S3: Correlations within groups separately.

NT

|  | ASRS | C4 ampl. Self | C4 ampl. Other |
| --- | --- | --- | --- |
| Touch Sensitivity | r=0.269  p=0.313 | r=-0.07  p=0.781 | r=-0.122  p=0.628 |
| C4 ampl. Self | r=0.074  p=0.763 |  |  |
| C4 ampl. Other | r=-0.313  p=0.191 |  |  |

ADHD

|  | ASRS | C4 ampl. Self | C4 ampl. Other |
| --- | --- | --- | --- |
| Touch Sensitivity | r=0.345  p=0.116 | r=0.387  p=0.066 | r=0.291  p=0.168 |
| C4 ampl. Self | r=-0.237  p=0.254 |  |  |
| C4 ampl. Other | r=0.149  p=0.478 |  |  |
